# The Advisory Committee on Immunization Practices’ Updated Interim Recommendation for Allocation of COVID-19 Vaccine — United States, December 2020

**DOI:** 10.15585/mmwr.mm695152e2

**Published:** 2021-01-01

**Authors:** Kathleen Dooling, Mona Marin, Megan Wallace, Nancy McClung, Mary Chamberland, Grace M. Lee, H. Keipp Talbot, José R. Romero, Beth P. Bell, Sara E. Oliver

**Affiliations:** ^1^CDC COVID-19 Response Team; ^2^Epidemic Intelligence Service, CDC; ^3^General Dynamics Information Technology, Falls Church, Virginia; ^4^Stanford University School of Medicine, Stanford, California; ^5^Vanderbilt University School of Medicine, Nashville, Tennessee; ^6^Arkansas Department of Health; ^7^University of Washington, Seattle, Washington.

*On December 22, 2020, this report was posted online as an *MMWR* Early Release.*

The first vaccines for prevention of coronavirus disease 2019 (COVID-19) in the United States were authorized for emergency use by the Food and Drug Administration (FDA) (*1*) and recommended by the Advisory Committee on Immunization Practices (ACIP) in December 2020.* However, demand for COVID-19 vaccines is expected to exceed supply during the first months of the national COVID-19 vaccination program. ACIP advises CDC on population groups and circumstances for vaccine use.^†^ On December 1, ACIP recommended that 1) health care personnel^§^ and 2) residents of long-term care facilities^¶^ be offered COVID-19 vaccination first, in Phase 1a of the vaccination program (*2*). On December 20, 2020, ACIP recommended that in Phase 1b, vaccine should be offered to persons aged ≥75 years and frontline essential workers (non–health care workers), and that in Phase 1c, persons aged 65–74 years, persons aged 16–64 years with high-risk medical conditions, and essential workers not recommended for vaccination in Phase 1b should be offered vaccine.** These recommendations for phased allocation provide guidance for federal, state, and local jurisdictions while vaccine supply is limited. In its deliberations, ACIP considered scientific evidence regarding COVID-19 epidemiology, ethical principles, and vaccination program implementation considerations. ACIP’s recommendations for COVID-19 vaccine allocation are interim and might be updated based on changes in conditions of FDA Emergency Use Authorization, FDA authorization for new COVID-19 vaccines, changes in vaccine supply, or changes in COVID-19 epidemiology.

Since June 2020, ACIP has convened 10 public meetings to review evidence-based information pertaining to COVID-19 vaccines, including initial allocation of COVID-19 vaccine supplies.^††^ To inform policy options for ACIP, the COVID-19 Vaccines Work Group, comprising experts in infectious diseases, vaccinology, vaccine safety, public health, and ethics, held 28 meetings to review data regarding vaccine candidates, COVID-19 surveillance, modeling of allocation scenarios, and vaccination program implementation issues. The Work Group also considered the relevant scientific literature, including ethical principles related to vaccine allocation in the setting of limited supply. Following ACIP’s interim recommendation for vaccine allocation in Phase 1a (*2*), the Work Group proposed vaccine allocation for Phases 1b and 1c. A description of the population groups in these phases, supporting scientific data, consideration of ethical principles, and considerations for vaccination program implementation are presented in this report, and supporting evidence is available at https://www.cdc.gov/vaccines/hcp/acip-recs/vacc-specific/covid-19/evidence-table-phase-1b-1c.html.

## Phase 1b

Approximately 49 million persons, including frontline essential workers (non–health care workers) and persons aged ≥75 years are recommended to receive vaccine in Phase 1b of the COVID-19 vaccination program (Table). Essential workers perform duties across critical infrastructure sectors and maintain the services and functions that U.S. residents depend on daily. The Cybersecurity and Infrastructure Security Agency (CISA) of the U.S. Department of Homeland Security has developed a list intended to guide jurisdictions in identifying essential critical infrastructure workers, who may be exempted during stay-at-home-orders (*3*). ACIP used CISA guidance to define frontline essential workers as the subset of essential workers likely at highest risk for work-related exposure to SARS-CoV-2, the virus that causes COVID-19, because their work-related duties must be performed on-site and involve being in close proximity (<6 feet) to the public or to coworkers. ACIP has classified the following non–health care essential workers as frontline workers: first responders (e.g., firefighters and police officers), corrections officers, food and agricultural workers, U.S. Postal Service workers, manufacturing workers, grocery store workers, public transit workers, and those who work in the education sector (teachers and support staff members) as well as child care workers.^§§^ A tiered approach for essential workers builds on the occupations identified by the National Academies of Science, Engineering and Medicine for early vaccination (*4*).

**TABLE:** Advisory Committee on Immunization Practices recommendations for allocation of COVID-19 vaccines to persons aged ≥16 years — United States, December 2020

Phase	Groups recommended to receive COVID-19 vaccine	No. (millions)		
		Total persons in each group*	Unique persons in each group^†^	Unique persons in each phase
1a	Health care personnel	21	21	24
	Long-term care facility residents	3	3	
1b	Frontline essential workers^§^	30	30	49
	Persons aged ≥75 years	21	19	
1c	Persons aged 65–74 years	32	28	129
	Persons aged 16–64 years^¶^ with high-risk medical conditions	110	81	
	Essential workers^§^ not recommended for vaccination in Phase 1b	57	20	
2	All persons aged ≥16 years^¶^ not previously recommended for vaccination	All remaining	All remaining	All remaining

**Abbreviation:** COVID-19 = coronavirus disease 2019.

* Data sources for each group: health care personnel (American Community Survey, 2019; https://www.census.gov/programs-surveys/acs/data.html); long-term care facility residents (Minimum Data Set. Centers for Medicare & Medicaid Services; https://data.cms.gov/); frontline and other essential workers (American Community Survey, 2019; https://www.census.gov/programs-surveys/acs/data.html); age-specific groups (U.S. Census; https://data.census.gov/cedsci/); high-risk medical conditions (Behavioral Risk Factors Surveillance System, 2018; https://www.cdc.gov/brfss/annual_data/annual_data.htm).

^†^ Excludes persons who were recommended to receive vaccine in an earlier phase (e.g., persons aged 65–74 years who are living in long-term care facilities or who are health care personnel, who would have been included in Phase 1a) and accounting for overlap between groups within the same phase (e.g., essential workers with high risk medical conditions).

^§^ Estimates for frontline and other essential workers are approximate and derived from prepandemic survey data; relative proportions will vary by state.

^¶^ As of December 18, only the Pfizer-BioNTech COVID-19 vaccine is authorized for use in persons aged 16–17 years.

Although there is no national surveillance for COVID-19 among frontline or other essential workers, reports of high incidence and outbreaks within multiple critical infrastructure sectors illustrate the COVID-19 risk in these populations and the disproportionate impact of COVID-19 on workers who belong to racial and ethnic minority groups. During March–June, for example, the Utah Department of Heath reported 1,389 COVID-19 cases associated with workplace outbreaks in 15 industry sectors, accounting for 12% of all COVID-19 cases in Utah during the same period (*5*). In addition, despite representing 24% of Utah workers in all affected sectors, Hispanic and non-White workers accounted for 73% of COVID-19 cases in workplace-associated outbreaks (*5*). Among 23 states reporting COVID-19 outbreaks in meat and poultry processing facilities during April and May, 16,233 outbreak-associated cases were reported from 239 facilities, including 86 COVID-19–related deaths (*6*). The percentage of workers with COVID-19 ranged from 3% to 25% per facility, and among cases with information on race and ethnicity reported, 87% occurred among workers from racial or ethnic minority groups (*6*).

Persons aged ≥75 years are at high risk for COVID-19–associated morbidity and mortality. As of December 20, 2020, the cumulative incidence^¶¶^ of COVID-19 among persons in this age group was 3,839 per 100,000 persons, with a cumulative hospitalization rate of 1,211 per 100,000, and a mortality rate of 719 per 100,000 (*7*–*9*). The overall proportion of persons aged ≥75 years who live in a multigenerational household is 6%; the proportion among non-Hispanic White persons is 4%, and the proportion among racial or ethnic minority groups is higher (non-Hispanic Black persons, 10%; Hispanic or Latino persons, 18%; non-Hispanic persons of other races, 20%).***

## Phase 1c

In Phase 1c, vaccine should be offered to persons aged 65–74 years, persons aged 16–64 years^†††^ with medical conditions that increase the risk for severe COVID-19, and essential workers not previously included in Phase 1a or 1b. Approximately 129 million persons are included in Phase 1c (Table), accounting for the overlap between groups in Phase 1c and earlier phases; for example, some adults aged 65–74 years reside in long-term care facilities, and many essential workers have high-risk medical conditions. Persons aged 65–74 years are at high risk for COVID-19–associated morbidity and mortality. As of December 20, 2020, the cumulative COVID-19 incidence in this age group was 3,109 per 100,000 persons, with a cumulative hospitalization rate of 642 per 100,000, and a mortality rate of 188 per 100,000 (*7*–*9*).

Based on ongoing review of the literature, CDC has identified medical conditions or risk behaviors that are associated with increased risk for severe COVID-19.^§§§^ The risk for COVID-19–associated hospitalization increases with the number of high-risk medical conditions, from 2.5 times the risk for hospitalization for persons with one condition to 5 times the risk for those with three or more conditions (*10*). According to a recent analysis of 2018 Behavioral Risk Factor Surveillance System data,^¶¶¶^ at least 56% of persons aged 18–64 years report at least one high-risk medical condition (CDC COVID-19 Response Team, Division of Population Health, personal communication, December 2020). Essential worker sectors recommended for vaccination in Phase 1c include those in transportation and logistics, water and wastewater, food service, shelter and housing (e.g., construction), finance (e.g., bank tellers), information technology and communications, energy, legal, media, public safety (e.g., engineers), and public health workers.****

ACIP’s ethical principles for allocating initial supplies of COVID-19 vaccine, namely, to maximize benefits and minimize harms, promote justice, and mitigate health inequities (*11*), support the allocation scheme for Phases 1b and 1c. Allocation of COVID-19 vaccine to essential workers and persons at increased risk for severe COVID-19 disease balances the vaccination program priorities of minimizing societal disruption and preventing morbidity and mortality. Essential workers constitute a large and heterogenous group. Allocation of vaccine to frontline essential workers in Phase 1b acknowledges their increased risk for occupational exposure compared with other essential worker categories, as well as the benefits to society of maintaining these essential functions. Allocation to persons aged ≥75 years is supported by their high risk for COVID-19–associated morbidity and mortality and is anticipated to also reduce hospitalizations in this group, easing the burden on strained health care systems. Populations included in Phase 1c are either at an increased risk for severe COVID-19 compared with the general population or support ongoing critical infrastructure operations. In addition, certain essential worker groups have high proportions of some racial and ethnic minority groups who have experienced disproportionate COVID-19 incidence, morbidity, and mortality (*12*).

Implementing vaccination programs to reach essential workers will pose challenges. Use of multiple strategies is recommended to reduce barriers to vaccination,^††††^ such as providing vaccination opportunities at or close to the workplace. State and local health authorities will need to take local COVID-19 epidemiology and demand for vaccine into account when deciding to proceed to the next phase or to subprioritize within an allocation phase if necessary. A flexible approach to allocation will facilitate efficient management and ensure that COVID-19 vaccine is administered equitably and without delay. Additional interim considerations for phased implementation of COVID-19 vaccines are available at https://www.cdc.gov/vaccines/covid-19/initial-populations.html and https://www.cdc.gov/vaccines/covid-19/phased-implementation.html.

## Phase 2

Phase 2 includes all other persons aged ≥16 years not already recommended for vaccination in Phases 1a, 1b, or 1c. Currently, in accordance with recommended age and conditions of use (*1*), any authorized COVID-19 vaccine may be used. ACIP is closely monitoring clinical trials in children and adolescents and will consider recommendations for use when a COVID-19 vaccine is authorized for use in persons aged <16 years.

**Summary**

**What is already known about this topic?**

On December 1, the Advisory Committee on Immunization Practices (ACIP) recommended that health care personnel and long-term care facility residents be offered COVID-19 vaccination first (Phase 1a).

**What is added by this report?**

On December 20, ACIP updated interim vaccine allocation recommendations. In Phase 1b, COVID-19 vaccine should be offered to persons aged ≥75 years and non–health care frontline essential workers, and in Phase 1c, to persons aged 65–74 years, persons aged 16–64 years with high-risk medical conditions, and essential workers not included in Phase 1b.

**What are the implications for public health practice?**

Federal, state, and local jurisdictions should use this guidance for COVID-19 vaccination program planning and implementation.

## Footnotes

* https://www.cdc.gov/vaccines/hcp/acip-recs/vacc-specific/covid-19.html.

† https://www.cdc.gov/vaccines/acip/committee/acip-charter.pdf.

§ https://www.cdc.gov/infectioncontrol/guidelines/healthcare-personnel/appendix/terminology.html.

¶ https://www.cdc.gov/longtermcare/index.html.

** On December 20, 2020, ACIP voted 13 to 1 in favor of the Phase 1b and 1c allocation recommendations.

†† https://www.cdc.gov/vaccines/acip/meetings/index.html.

§§ https://www.cdc.gov/vaccines/covid-19/categories-essential-workers.html.

¶¶ Incidences were calculated using age-specific population denominators from the U.S. Census. https://www.census.gov/data.html.

*** Data from the U.S. Census Bureau 2019 American Community Survey 1-Year Public Use Microdata Samples [CSV file]. https://www2.census.gov/programs-surveys/acs/data/pums/2019/1-Year/. Accessed December 16, 2020.

††† As of December 18, 2020, two COVID-19 vaccines have been authorized for use under an Emergency Use Authorization (EUA), but only the Pfizer-BioNTech COVID-19 vaccine is authorized for use in persons aged 16–17 years.

§§§ Adults of any age with the following conditions are at increased risk for severe COVID-19–associated illness: cancer; chronic kidney disease; chronic obstructive pulmonary disease (COPD); heart conditions, such as heart failure, coronary artery disease, or cardiomyopathies; immunocompromised state (weakened immune system) from solid organ transplant; obesity (body mass index [BMI] ≥30 kg/m^2^ but <40 kg/m^2^); severe obesity (BMI ≥40 kg/m^2^); sickle cell disease; smoking; type 2 diabetes mellitus; and pregnancy. No data are currently available on the safety of COVID-19 vaccines in pregnant persons. If pregnant persons are part of a group that is recommended to receive a COVID-19 vaccine (e.g., health care personnel or essential worker), they may choose to be vaccinated. A conversation between the patient and the patient’s clinical team might assist with decisions regarding the use of vaccines approved under EUA for the prevention of COVID-19. Guidance for pregnant persons will be updated as new data becomes available at https://www.cdc.gov/vaccines/covid-19/info-by-product/clinical-considerations.html. The list of high-risk medical conditions is updated routinely as new data becomes available at https://www.cdc.gov/coronavirus/2019-ncov/need-extra-precautions/people-with-medical-conditions.html.

¶¶¶ https://www.cdc.gov/brfss/annual_data/annual_data.htm.

**** Certain occupations in Phase 1b might be related to sectors listed in Phase 1c (public transit [transportation and logistics], grocery store workers [food services], and corrections workers [public safety]).

†††† COVID-19 Vaccination Communication Toolkit. https://www.cdc.gov/vaccines/covid-19/health-systems-communication-toolkit.html.
